# Intracrine Endorphinergic Systems in Modulation of Myocardial Differentiation

**DOI:** 10.3390/ijms20205175

**Published:** 2019-10-18

**Authors:** Silvia Canaider, Federica Facchin, Riccardo Tassinari, Claudia Cavallini, Elena Olivi, Valentina Taglioli, Chiara Zannini, Eva Bianconi, Margherita Maioli, Carlo Ventura

**Affiliations:** 1 National Laboratory of Molecular Biology and Stem Cell Bioengineering - Eldor Lab, National Institute of Biostructures and Biosystems (NIBB), at the Innovation Accelerator, CNR, Via Piero Gobetti 101, 40129 Bologna, Italy; silvia.canaider@unibo.it (S.C.); federica.facchin2@unibo.it (F.F.); riccardo.tassinari.rt@gmail.com (R.T.); clo.cavallini@gmail.com (C.C.); elecorte82@gmail.com (E.O.); valentina.taglioli3@unibo.it (V.T.); chiara.zannini5@unibo.it (C.Z.); eva.bianconi2@gmail.com (E.B.); 2 Department of Experimental, Diagnostic and Specialty Medicine (DIMES), University of Bologna, Via Massarenti 9, 40138 Bologna, Italy; 3 Department of Biomedical Sciences, University of Sassari, Viale San Pietro 43/B, 07100 Sassari, Italy; mmaioli@uniss.it

**Keywords:** stem cells, dynorphin B, *prodynorphin* gene, intracrine, nuclear opioid receptors, transcription factors, cardiogenesis, cardiac regeneration, hyaluronan esters, electromagnetic fields

## Abstract

A wide variety of peptides not only interact with the cell surface, but govern complex signaling from inside the cell. This has been referred to as an “intracrine” action, and the orchestrating molecules as “intracrines”. Here, we review the intracrine action of dynorphin B, a bioactive end-product of the *prodynorphin* gene, on nuclear opioid receptors and nuclear protein kinase C signaling to stimulate the transcription of a gene program of cardiogenesis. The ability of intracrine dynorphin B to prime the transcription of its own coding gene in isolated nuclei is discussed as a feed-forward loop of gene expression amplification and synchronization. We describe the role of hyaluronan mixed esters of butyric and retinoic acids as synthetic intracrines, controlling *prodynorphin* gene expression, cardiogenesis, and cardiac repair. We also discuss the increase in *prodynorphin* gene transcription and intracellular dynorphin B afforded by electromagnetic fields in stem cells, as a mechanism of cardiogenic signaling and enhancement in the yield of stem cell-derived cardiomyocytes. We underline the possibility of using the diffusive features of physical energies to modulate intracrinergic systems without the needs of viral vector-mediated gene transfer technologies, and prompt the exploration of this hypothesis in the near future.

## 1. Introduction

Cell-to-cell communication is usually viewed as a signaling cross-talk between neighboring cells, referred to as paracrine communication, or as a modality in which a given cell is able to release signaling molecules that in turn bind receptors on that same cell, according to a so-called autocrine communication.

**Figure 1:**
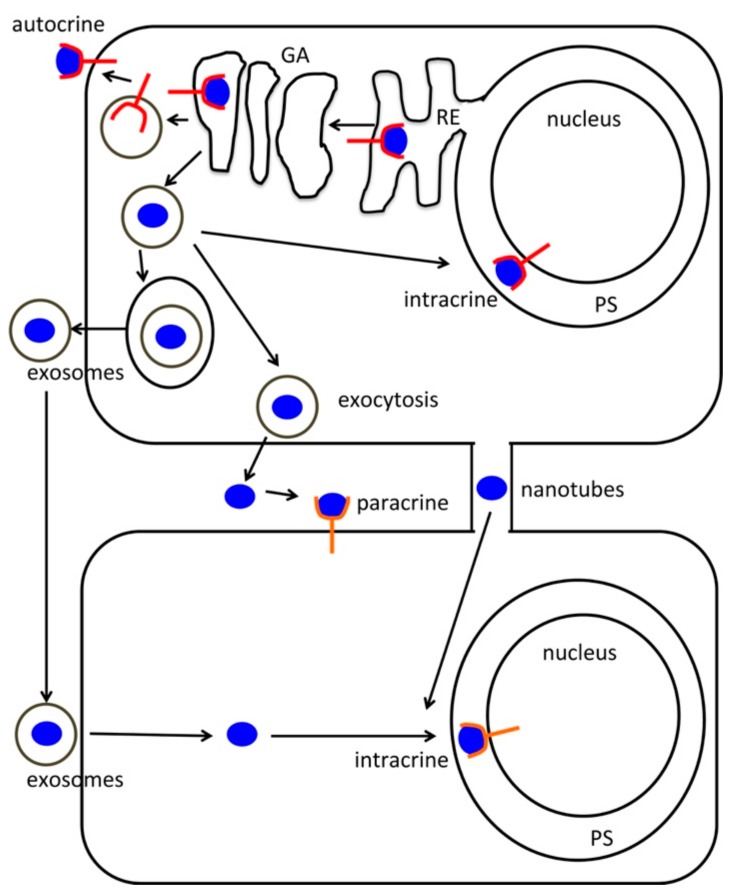
Intracrine patterning. The figure depicts a scheme of intracrine signaling within the context of intra- and extra-cellular communication via paracrine, autocrine, and exosomal routes. GA: Golgi Apparatus; RE: Endoplasmic Reticulum; PS: Perinuclear Space; red shape: receptor; blue shape: signal.

In 1984, Re and Coworkers introduced the term intracrine, to define a peptide action within the cell interiors, identifying a different route as compared to a peptide/hormone acting at the level of cell-surface receptors [1,2]. An intracrine could, therefore, then be defined as an agonist, including a hormone or other signaling peptides/proteins, controlling cellular dynamics from within the cell of synthesis, or inside a target cell after internalization [3,4]. The notion of intracrine physiology grew up over time, generating novel perspectives in the way of conceiving intracellular trafficking and cell signaling. A remarkably growing number of endogenous molecules have been added to the intracrine list during the last few years, including hormones, cytokines, and many growth factors, whose action was believed to occur only at the plasma membrane level [4,5]. A significant breakthrough in the deployment of intracrine mechanisms came from the progressive awareness that most of the signaling players are not acting as naked molecules, but they can be rather travelling among and inside cells packaged within exosomes. The multifaceted content of these nanovesicles can be poured inside the cells as “pocket-of-information” controlling nuclear trafficking, epigenetic and transcriptional patterning. Consonant with this intriguing scenario, it is now evident that even transcription factors, DNA binding proteins, and enzymes can be exchanged through the exosomal route [6,7], and also likely through cellular nanotubes, a kind of nanostructures that are currently emerging as an additional modality of inter-/intra-cellular spreading of biological information [8,9,10]. The existence of nuclear and/or other intracellular binding sites capable of unfolding the presence of these molecules into concerted cell signaling pathways are now offering novel clues to reinterpret the role of nanovesicular/nanotubular transport systems. Nonetheless, the intracrine world is posing new challenges in deciphering the subtle line of demarcation between physiological and pathological patterns (Figure 1).

Growing evidence has accumulated over recent years showing that the biological effect of Angiotensin II on its target genes can be mediated by the interaction of Angiotensin II with intracellular receptor types 1 and 2 (AT_1_ and AT_2_), associated with intracrine responses [11]. In human mesangial cells both receptors were found in the nuclear membrane, and the addition of labeled Angiotensin II to isolated mesangial cell nuclei produced a fluorescence that could be inhibited by specific receptor antagonists [11]. Cell exposure to high glucose, which stimulates endogenous intracellular Angiotensin II synthesis was able to induce mesangial cell proliferation and overexpression of fibronectin even in the presence of candesartan which prevents Angiotensin II internalization, therefore, indicating an intracrine action of endogenous, high glucose-induced Angiotensin II, independent of cell surface receptors [11].

Vascular endothelial growth factor (VEGF) is another peptide playing a remarkable role in both somatic and stem cell dynamics. Hematopoietic stem cells (HSCs) express and secrete VEGF, and during their development to megakaryocytes (MKs) the structurally related receptors VEGFR1, VEGFR2, and VEGFR3 are expressed at a different developmental stage. VEGF has been shown to act in an intracrine fashion to promote HSC survival and repopulation [12,13]. Moreover, VEGFR2 has been found in the nucleus of human erythroleukemia cells (HEL), with features of MKs, being constitutively phosphorylated [14], and could be inhibited by internal VEGFR2-specific inhibitor, to prevent constitutive activation of MAPK/ERK and PI3/AKT, therefore, leading to HEL apoptosis [14]. Conversely, extracellular acting anti VEGF monoclonal antibody only elicited a weak apoptotic response [14]. These findings indicate: (1) The relevance of the intracrine pathway in HSC dynamics; (2) the fact that autocrine/paracrine, and intracrine loops, as those mediated by VEGF, act by modulating different stem cell functions and signaling pathways; (3) the complexity of the intracrine loops, and that fact that a subtle demarcation line divides the beneficial effects of intracrine peptides from the pathological outcomes resulting from malfunctioning of their related pathways. Accordingly, VEGF-VEGFR intracrine signaling has been shown to be required for both normal endothelial cell homeostasis and tumor-associated endothelial cell survival [15,16]. It has also been recently reported that a dysregulation of intracrine VEGF-VEGFR axis within the control of apoptotic, survival, and proliferation pathway may also lead to the survival and proliferation of unwanted cell types, as it has been shown for colorectal cancer [17], as well as in normal and pathological (i.e., malignant) developmental stages of MKs, and platelets [18,19,20,21]. 

The list of peptides acting not only in a paracrine/autocrine, but even intracrine fashion is fated to grow exponentially, as shown by the dissection of the intracrine action of Fibroblast Growth Factor 1 (FGF1), a prototypic member in the FGFs family, whose intracrine modality of action has been shown to be executed at nuclear level to induce neuronal differentiation and regulate stem cell response to apoptosis. In this regard, the induction of mutation in specific domains of FGF1 has provided remarkable insights in elucidating its nuclear pathways. Overall, there is now compelling indication that future studies on the intracrine responses mediated by peptides subjected to targeted domain mutations may provide significant breakthrough on how intracrine peptides may affect normal stem cell differentiation, or they may be conversely responsible for malignant stem cell transformation, tumor dissemination and resistance to radio- and chemo-therapy [22]. Akin to this perspective, photo-releasable ligands [23], and caged ligands [24] are currently under development as additional tools to study the intracrine signaling of various peptides in intact cells.

Compounding the complexity of intracrinergic systems, a novel cardiac intracrine mechanism has been recently shown in the heart, where Angiotensin-(1–12), a newly uncovered angiotensinogen derivative, is acting as the precursor of Angiotensin II, and a chymase rather than the Angiotensin Converting Enzyme (ACE) is the main enzyme involved in the production of Angiotensin II from Angiotensin-(1–12) [25]. It is now evident that the Angiotensin-(1–12)/chymase axis represents an independent intracrinergic system accounting for major effects exerted by Angiotensin II in the human heart, including trophic, contractile and rhythmic dynamics [25]. There is now evidence that dysregulation of this intracrine axis may play a major role in the onset and progression of human heart failure, as suggested by the finding that Angiotensin-(1–12) and chymase expression and activity are both elevated in the heart of patients undergoing heart failure [25]. These findings, accounting for the production of Angiotensin II independently from the circulatory Renin/Angiotensin System, may also explain the shortcomings of ACE inhibition and Angiotensin receptor blockade in a consistent number of patients, who, differently from the one responding to this treatment, will remain at risk for heart disease progression.

Within the context of the cardiovascular system, it is now emerging that intracrine physiology may further unfold the basis for understanding the exchange of chemical signals between cardiac cells. In these cells, the role of gap junctions in transferring chemical information can now be viewed in close association with the intracellular action of different factors (hormones, regulatory peptides) acting through an intracrine modality to affect cell volume and shape, chemical coupling and cardiac energetics [26]. The intracrine view building around the heart may help to conceive this organ as a metabolic syncytium, where glucose, nucleotides and hormones can be engaged in cell-to-cell trafficking through gap junctions [26]. This view may help to provide a new vision on how cardiac intracrine pathways may be part of a “fruitful” cooperative scenario under physiological conditions, or they may undergo unbalanced interplay, drifting from cardiac physiology to cardiac pathological states.

On the basis of these premises, we here intended to review the experimental works that investigate the intracrine role of Dynorphin B in stem and myocardial cells and the modulation/expression of this endorphin following hyaluronan mixed esters of butyric and retinoic acids (HBR) or electromagnetic energy administration. We retain that the study of intracrinergic pathways at the stem cell level may help to understand the mechanisms of cardiogenesis better, and provide potential new strategies in cardiac repair and regeneration.

## 2. Opioid Peptides and the Intracrine Regulation of Cellular Dynamics: A Pattern Highly Oriented Towards Stem Cell Cardiogenesis

**Figure 2:**
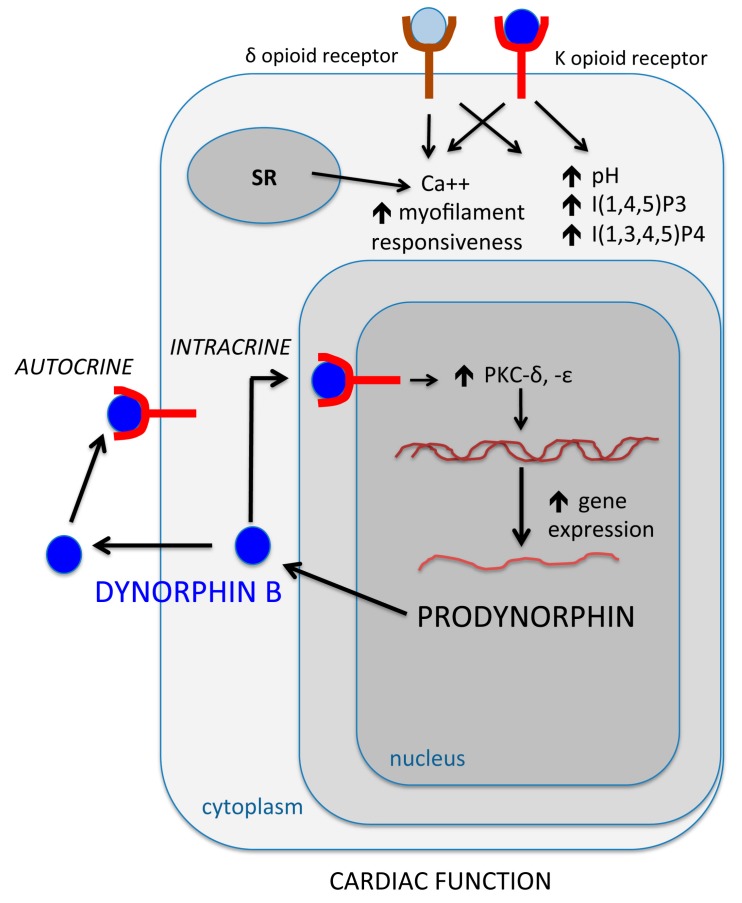
Intracrine dynorphin B pathway in rat ventricular cardiomyocytes. Th figure represents a virtual cell where different experimental evidence (detailed in the manuscript) is schematically shown. SR: Sarcoplasmic reticulum; Ins(1,3,4,5)P4: Inositol 1,3,4,5-tetrakisphosphate; Ins(1,4,5)P3: Inositol 1,4,5-trisphosphate; PKC: Protein kinase C.

Opioid peptides (also referred to as endorphin peptides) are co-released with other neurotransmitters from both sympathetic and parasympathetic nerve endings, acting at presynaptic level to inhibit noradrenaline [27,28,29] or acetylcholine release [30,31,32,33,34]. It is now established that at myocardial level endorphin peptides not only act by modulating the neurotransmitter release from the nerve endings supplying the heart [35,36,37,38,39], but they can also directly act on specific receptors that have been detected on myocardial cells. We provided evidence that among the three main opioid receptors, µ, δ and κ, both δ- and κ-opioid receptors were expressed in the sarcolemma of myocardial cells [40]. Moreover, a marked increase in selective agonist binding could be achieved in the presence of the α-adrenoceptor agonist phenylephrine or with the β-adrenoceptor agonist isoproterenol, indicating functional responsiveness of the identified binding sites. These observations prompted us to hypothesize that opioid receptors may be involved in the regulation of myocardial cell function. This perspective was then supported by a number of interrelated observations, showing that in cardiac myocytes κ and δ opioid receptor stimulation: (1) Increased cytosolic pH and myofilament responsiveness to Ca^2^; (2) affected phosphoinositide turnover by increasing the formation of both inositol 1,4,5-trisphosphate (Ins(1,4,5)P3), and inositol 1,3,4,5-tetrakisphosphate (Ins(1,3,4,5)P4) [41]; and (3) affected contractile dynamics in cardiomyocytes, as well as Ca^2+^ release from an intracellular pool in both cardiomyocytes and neurons [42,43]. These data, and other reports from studies showing that endorphins affected the contractility in isolated rat hearts [44,45], in dog myocardium [46], or cultured chick embryo heart cells [47,48], supported the notion that the myocardial cell could be a direct target for the action of these peptides (Figure 2). Even more intriguingly, large amounts of preproenkephalin mRNA, encoding for a peptide (preproenkephalin A), containing four copies of [Met]enkephalin and one copy each of [Leu]enkephalin, [Met]enkephalin-Arg6-Gly7-Leu8, and [Met]enkephalin-Arg6-Phe7, all mainly acting on δ opioid receptors, were initially detected in the rat heart tissue [49,50], that was found to contain greater amounts of the coding mRNA than any other rat tissue, including the brain [49]. Nevertheless, despite the large amounts of preproenkephalin mRNA in the heart, the cardiac content of the opioid peptide (preproenkephalin A) was only 3% the amount detected in the brain [49]. Concomitantly, remarkable amounts of prodynorphin-derived peptides, which are encoded by the *prodynorphin* gene, and mainly act as endogenous κ opioid receptor agonists, were found in human, rat, rabbit, and guinea-pig hearts [51]. These studies were conducted in multicellular preparations and could not discern the cell type responsible for the observed peptide synthesis. These findings induced us to investigate whether the *prodynorphin* gene and its related peptides may be expressed in the myocardial cell, and whether in the affirmative, the cardiac myocyte itself may be considered not only a target, but a source for endorphin peptides. This hypothesis was confirmed in subsequent studies showing that: (1) The *prodynorphin* gene was expressed in adult rat ventricular cardiomyocytes [52]; (2) dynorphin B, a bioactive end-product of the gene, could be detected both intracellularly and in the culture medium [52]; (3) the *prodynorphin* gene transcription, as well as the amount of intracellular and secreted dynorphin B, were enhanced by cardiomyocyte exposure to high potassium chloride (KCl) [52]; (4) the myocardial expression of the *prodynorphin* gene and dynorphin B (both the intracellular and secreted peptide) could also be enhanced by cell exposure to phorbol 12-myristate 13-acetate (PMA) through a mechanism depending upon the activation of nuclear protein kinase C (PKC) [53]; (5) the transcription of the *prodynorphin* gene was increased both in nuclei isolated from PMA-treated cardiomyocytes and in isolated myocardial nuclei directly treated with the phorbol ester [53]; (6) both PKC-δ and –ε were expressed in isolated myocardial nuclei, and the PKC inhibitor staurosporine abolished the increase in *prodynorphin* gene transcription elicited by the nuclear exposure to PMA [53] (Table 1). An additional consequence of these findings was that they provided the underpinning for the investigation of an intracellular modality of action of cardiac endorphins in the regulation of myocardial cell dynamics. In 1998 we discovered the presence of κ-opioid receptors in highly purified nuclei isolated from myocardial cells [54]. These studies revealed a significant increase in the maximal binding capacity for a selective κ-opioid receptor ligand in myocardial nuclei that had been isolated from BIO 14.6 cardiomyopathic hamsters, in comparison with nuclei obtained from normal cardiomyocytes of the F1B strain. Interestingly, the exposure of nuclei isolated from normal myocardial cells to dynorphin B, a natural agonist of κ-opioid receptors, remarkably enhanced the transcription of the *prodynorphin* gene [54]. This transcriptional response was mediated by nuclear PKC activation and occurred at a higher rate in nuclei obtained from cardiomyopathic myocytes than in nuclei that had been isolated from normal cardiomyocytes [54]. These initial studies provided the first evidence that a nuclear endorphinergic system was part of an intracrine loop involving a feed-forward stimulation of dynorphin B on the transcription of its own coding gene, imparting features of long-lived signaling and memory (Figure 2). 

**Figure 3:**
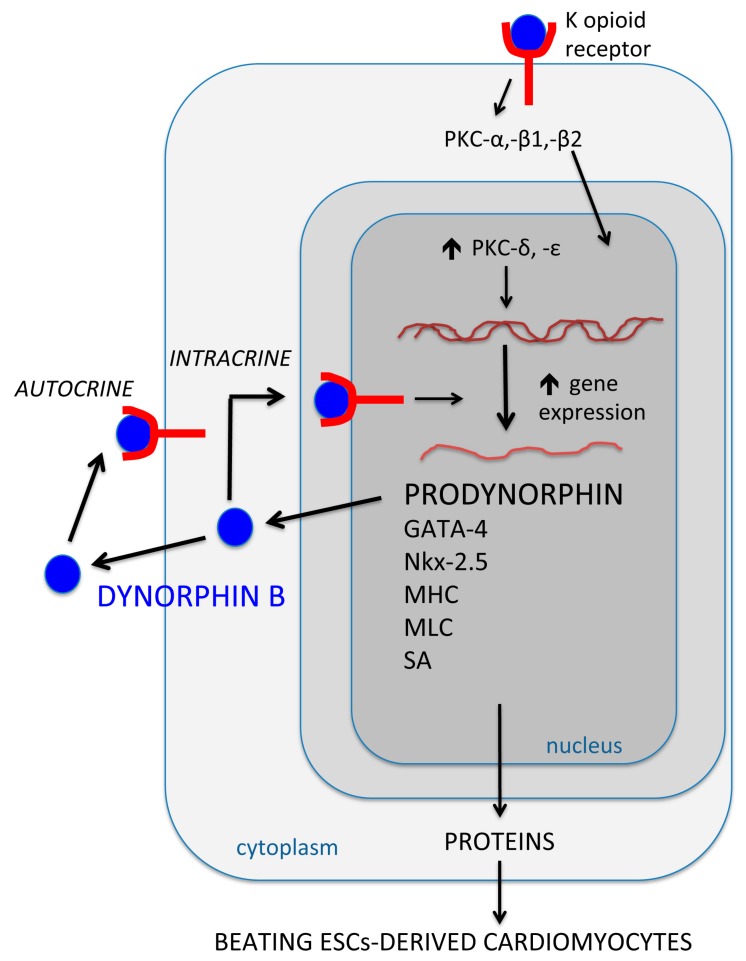
Intracrine dynorphin B pathway in embryonic stem cells (ESCs). The figure represents a virtual ES cell where different experimental evidence (detailed in the manuscript) is shown. MHC: α-Myosin heavy chain; MLC: Myosin light chain; SA: α-sarcomeric actinin.

In subsequent studies, the *prodynorphin* gene and its bioactive end-product dynorphin B emerged as major conductors of cardiogenesis in embryonic stem cells (ESCs) [55]. In pluripotent P19 cells, the transcription of *GATA-4* and *Nkx-2.5*, governing crucial developmental steps towards cardiogenesis, was induced by cell exposure to dimethyl sulfoxide (DMSO), and was preceded by a marked increase in *prodynorphin* gene expression. This transcriptional response was associated with a pronounced rise in the intracellular level of dynorphin B, indicating the chance for an intracrine action of the peptide in eliciting the cardiogenic process [55]. In DMSO treated cells, we also detected an increase of dynorphin B in the culture medium, suggesting the establishment of an autocrine mechanism [55] (Table 1), as we previously observed in cardiomyocytes of Syrian cardiomyopathic Hamsters [56,57,58], where a specific κ-opioid receptor antagonist was able to counteract the spontaneous overexpression of the *prodynorphin* gene and dynorphin B by interrupting an autocrine feed-forward loop sustained by the secreted peptide [58]. Interestingly, even in the absence of DMSO, dynorphin B-conditioned medium was able to trigger *GATA-4* and *Nkx-2.5* gene transcription, enhancing the gene and protein expression of α-myosin heavy chain (α-MHC) and myosin light chain (MLC)-2V, two markers of terminal cardiac differentiation [55]. A causal role of dynorphin B in ESC cardiogenesis was also inferred by the finding that the differentiating effect of DMSO could be counteracted by opioid receptor antagonism or targeted inhibition of *prodynorphin* gene expression [55]. These findings were further supported by the possibility to create a cardiogenic stem cell line based upon lentiviral mediated overexpression of the *prodynorphin* gene in mouse ESCs [59]. Lentiviral transduction enhanced the transcription of the cardiogenic genes *GATA-4* and *Nkx-2.5*, along with other markers of terminal differentiation, as α-sarcomeric actinin (SA), cardiac L-type Ca^2+^ channel, connexin 43, and dramatically increased the yield of spontaneously beating ESC-derived cardiomyocytes [59]. A compelling evidence for a major role of an endorphinergic system in cardiogenesis was provided by a number of interrelated findings showing that: (1) Endogenous dynorphin B was essential in the cardiogenic process of GTR1 ESCs, a derivative of mouse R1 ES cells bearing the puromycin resistance gene driven by the cardiomyocyte-specific α-MHC promoter [60]; (2) immunoreactive dynorphin B was faintly detected in undifferentiated ESCs, while being highly enhanced inside ESC-derived cardiomyocytes, gathering around the nucleus [61]; (3) κ-specific opioid binding sites, besides being expressed on ESC plasma membranes [60], could also be identified in ESC nuclei, with a single dissociation constant in the low-nanomolar range [61]; (4) a significant increase in the maximal binding capacity (Bmax) for a selective κ-opioid receptor ligand was detected in nuclei that had been isolated from ESC-derived cardiomyocytes, compared with nuclei from undifferentiated cells [61]. On this basis, we were further encouraged to understand whether the presence of nuclear endorphin receptors in ESCs may have represented the interface for a causative intracrine patterning in cardiogenesis. This hypothesis was confirmed by the finding that direct exposure to dynorphin B or U-50,488H, a synthetic κ-opioid receptor agonist, of nuclei isolated from undifferentiated ESCs was able to induce time- and dose-dependent increase in the transcription rate of both *GATA-4* and *Nkx-2.5* [61]. The incubation of ESC nuclei in the presence of dynorphin B also enhanced the rate of transcription of the *prodynorphin* gene, further highlighting the capability of this intracrine endorphinergic system to operate in a feed-forward fashion to upregulate its own synthesis or boost elements of its signaling cascade [61]. To this end, the intracrine action of dynorphin B on its nuclear receptors proved to be specific in nature, since besides being blocked by naloxone, a broad opioid receptor antagonist, it was also abrogated by Nor-binaltorphimine and Mr-1452, two selective κ-opioid receptor antagonists [61]. In this study, the observation that both Mr-1453 and (+) naloxone, two inactive enantiomers, failed to affect the transcriptional responses induced by dynorphin B, proved that its intracrine modality was exploited in a stereospecific fashion on the identified nuclear opioid receptors. The finding that exposure of ESC nuclei to dynorphin B failed to affect the transcription of both *MyoD* and *neurogenin-1*, involved in skeletal myogenesis and neurogenesis, respectively, provides evidence that the intracrine action of dynorphin B retained an extremely high degree of selectivity, being essentially oriented to a cardiogenic decision [61]. In the attempt of elucidating the signaling pathways triggered by intracrine dynorphin B, we started from our previous studies showing that PKC signaling was able to transduce the cardiogenesis primed by endogenous dynorphin B in ESCs, and that cardiac differentiation was associated with nuclear translocation of PKC-α, -β1, and -β2, while the expression of PKC-δ and -ε was mainly restricted to ESC nuclei [24]. Both PKC-δ and -ε resulted in being overexpressed in nuclei of ESC-derived cardiomyocytes, and their increase occurred independently of translocation, likely as a consequence of changes in isozyme (PKC-δ and -ε) turnover and/or gene expression. While κ-opioid receptor antagonists, acting on plasma membrane opioid receptors, prevented the nuclear translocation of PKC-α, -β1, and -β2, they failed to affect the nuclear increase in PKC-δ and -ε [61]. Notably, opioid receptor antagonism, by interfering with the autocrine loop mediated by secreted dynorphin B, reduced, but did not abolish the yield of spontaneously beating ESC-derived cardiomyocytes, indicating that an additional mechanism of cardiogenesis may have been occurred. The observation that cell permeant PKC inhibitors conversely caused a complete abrogation of cardiogenesis led us to investigate whether nuclear resident PKC isoforms, namely -δ, and -ε, may represent the missing circuit in the intracrine signaling cascade, linking the stimulation of nuclear opioid receptors to the observed transcription of cardiogenic genes. Confirming this hypothesis, nuclei isolated from undifferentiated ESCs were able to phosphorylate the acrylodan-labeled MARCKS peptide, a high-affinity fluorescent PKC substrate. Exposure of these nuclei to dynorphin B was able to induce a remarkable increase in nuclear PKC activity, which was suppressed by opioid receptor antagonists [61]. Worthy to note, PKC inhibitors not only suppressed the nuclear PKC activity, but abolished the transcriptional responses primed by dynorphin B in isolated nuclei (i.e., the induction of *GATA-4*, *Nkx-2.5*, and *prodynorphin* gene transcription) [61]. These findings, considered together with the observation that the same PKC inhibitors were able to abrogate the cardiac differentiation in ESCs, further supported the evidence that dynorphin B plays a major role in ESC cardiogenesis, and that nuclear opioid receptors and nuclear PKC were coupled to stem cell cardiac differentiation through the execution of an intracrine circuitry (Figure 3).

At the time of our discovery of nuclear endorphinergic pathways regulating prodynorphin and cardiogenic gene transcription, we hypothesized that newly synthesized dynorphin B or a partial amount of it, may react with opioid receptors in the perinuclear space, without being secreted extracellularly. Through the years, it has become evident that a consistent number of small peptides do not exert their effects as naked molecules, but they are trafficking among cells via an exosomal route. This may imply that a part of dynorphin B, packaged within exosomes, once secreted may use exosomes to enter the cells, then acting via an intracrine pathway following internalization. Detailed investigation of these issues is still lacking, and further studies are needed to elucidate whether exosomal trafficking of dynorphin B and other endorphin peptides may significantly take place and affect cellular dynamics.

The cardiogenic effect of dynorphin B has been recently confirmed in mice cardiac progenitor cells (CPCs), an Nkx-2.5 expressing cell population detectable in mid-gestation (E 11.5) mouse ventricles [62]. CPCs have fewer mitochondria than cardiomyocytes, and could be efficiently fractionated by the aid of a fluorescent dye targeting mitochondrial membrane potential, being separated by the cardiomyocyte population containing a greater number of mitochondria [62]. Despite the expression of Nkx-2.5, only a low fraction (about 1%) of the low-mitochondria CPCs can be positively stained with MF20, recognizing the expression of α-MHC. Exposure of CPCs to dynorphin B caused a remarkable increase in the number of CPC-derived cardiomyocytes [62], while exposure to the conditioned medium yielded from high-mitochondria fraction of cardiac myocytes resulted in significant inhibition of cardiogenesis, suggesting a paracrine inhibitory role of adult cardiomyocytes over the cardiogenic potential of CPCs [62]. Further studies are required to investigate the potential contribution of an intracrine pathway in the cardiogenesis induced by dynorphin B in CPCs. Dissecting the relative contribution of intracrine and paracrine/autocrine pathways in CPCs subjected to the treatment with dynorphin B alone, or in combination with other cardiac lineage promoting agents, may prove rewarding in optimizing the cardiogenic potential of CPCs and their engraftment efficiency in experimental models of acute myocardial infarction or chronic heart failure. 

On the whole, the above-reported observation should be taken into account when dissecting the implications that may arise from the use of dynorphin B knockout animals. In fact, initially established homozygous mutant mice lacking dynorphin B did not show any apparent impairment, compared to heterozygous and wild type littermates with respect to cage behavior, growth, weight, fertility, and longevity [63]. Nevertheless, subsequent investigation revealed that κ opioid receptor null mice suffered for altered bone morphogenesis [64], and accelerated cartilage degeneration after injury [65], in comparison to wild type animals, and that the associated defects could be efficiently rescued by κ opioid receptor activation [64,65]. Moreover, κ opioid receptor-null or dynorphin-null mice revealed a pronounced enhancement in overall vascular formation, associated with ectopic vascular invasion into somites at day 10.5 of embryonic development [66], indicating that dynorphin B knockout could also be used to unveil regulation of endothelial cell differentiation and pathfinding in vascular development by a κ opioid receptor system. These reports, together with other results providing wide-ranging regulation of metabolic and adaptive responses by dynorphinergic systems in dynorphin B knockout mice [67,68], suggest that given the complexity of heart development and of the associated process of cardiomyogenesis, further, more accurate studies are needed to explore the effect of dynorphin B and/or κ opioid receptor knockout on the expression of the cardiac lineage potential in targeted subset of stem cells, including CPCs. Addressing these issues may provide further insights into the potential involvement of paracrine/autocrine or intracrine patterning to explain the initial observation that high-level of endorphin gene expression could be detected in post-natal rat hearts [49,50], and that exposure to the opioid antagonist naltrexone throughout gestation was able to alter postnatal heart development [69], while dynorphin B administered to newborn rats was found to modulate heart morphogenesis [70].

## 3. Hyaluronan Mixed Esters of Butyric and Retinoic Acids (HBR): A Synthetic Intracrine Promoting Prodynorphin Gene and Dynorphin B Expression, Stem Cell Cardiogenesis and Cardiac Repair

Dynorphin B, like other endorphins, is an easily degradable, short-lived peptide, subjected to rapid inactivation by natural metallo-endopeptidases and aminopeptidases. An ideal strategy to afford a long-lasting intracellular availability of dynorphin peptides and sustain their intracrine action should, therefore, rely upon increasing the transcription of the *prodynorphin* gene, possibly without resorting to complex approaches of viral vector-mediated gene transfer technology. Within this context, we have developed HBR, with the aim of making available a synthetic cardiac/vascular lineage-promoting agent [71]. Consistent observations provided a rationale behind the development of HBR. The hyaluronan CD44 receptor is remarkably expressed by cardiac progenitors [72], and normal cardiogenesis can be suppressed by chemical inhibition of type-2 hyaluronan synthase [73]. Hyaluronan primes CD44-dependent endothelial cell differentiation [74], and CD44 plays a relevant role in vasculogenesis [75]. Hyaluronan can enter the cell through CD44 receptor-mediated endocytosis, and it has been found in close association with nuclear heterochromatin [76]. Hyaluronan-binding molecules (hyaladherins) can translocate to the nucleus upon mitogenic stimulation [77], serving as docking and activators for protein kinases and transcription factors that promote cellular growth and differentiation [78]. Therefore, we hypothesized that, while being taken up by the stem cells, hyaluronan could also be exploited as a carrier for the internalization of hyaluronan-grafted molecules essential in the cardiogenic process, such as butyric and retinoic acids. Butyric acid is capable of increasing transcription factor accessibility to targeted DNA regulatory sites, owing to histone deacetylase inhibition, and consequent hyperacetylation of nucleosomal histones [79]. Accordingly, in mouse ESCs, inhibition of histone deacetylase and shear stress-induced histone acetylation has been shown to elicit epigenetic patterns responsible for the activation of transcriptional signatures of cardiovascular commitment [80]. Concerning retinoic acid, its role in cardiovascular commitment is supported by the observation that abnormal cardiogenesis results from inactivation of the *RXRα* gene transcription, and from the combination of mouse strains with mutant RAR and RXR subtypes [81,82]. Moreover, the efficiency of cardiogenesis can be increased in ESCs by all-*trans* and 9-*cis*-retinoic acids [83]. Retinoic acid is also required in vasculogenesis in vivo [84], is essential in mammalian vascular development.

When assessed in mouse ESCs, HBR induced overexpression of the *prodynorphin* gene, together with an increase in *GATA-4* and *Nkx-2.5* transcription [71]. These transcriptional responses were already evident at the stage in which cells were grown as embryoid bodies in suspension and persisted in ESC-derived cardiomyocytes. Since from the early stage of HBR induced cardiac commitment, the intracellular level of dynorphin B was remarkably increased, suggesting the activation of an intracrine route of cardiogenesis [71]. In HBR treated cells, the intracellular amount of dynorphin B further increased throughout the acquirement of a terminally differentiated state, with a remarkably greater number of spontaneously beating myocardial cells, as compared to the untreated controls. The observed increase of intracellular dynorphin B in HBR exposed ESCs was paralleled by a consistent raise of peptide levels in the culture medium [71]. This observation suggests that secreted dynorphin B may also have enhanced the cardiogenic yield through the establishment of an autocrine mechanism, as it was previously shown to occur in the course of the spontaneous ESC cardiogenesis [60]. The cardiogenic/vasculogenic action observed with HBR in ESCs, could also be exploited in human mesenchymal stem cells (hMSCs) isolated from different sources, including the bone marrow, dental pulp, and fetal membranes of term placenta (FMhMSCs) [85]. In these cells, HBR enhanced *VEGF*, kinase insert domain receptor (*KDR*, a gene encoding a major VEGF receptor), and hepatocyte growth factor (*HGF*) gene expression, as well as the secretion of the angiogenic, mitogenic, and antiapoptotic factors VEGF and HGF, priming stem cell differentiation into endothelial cells [85]. Confirming the results yielded in ESCs, in multisource hMSCs, HBR increased both the transcription of *GATA-4* and *Nkx-2.5*, and the yield of cardiac marker-expressing cells [85]. These responses were notably more pronounced in FMhMSCs.

The fact the HBR was not able to affect the commitment of mouse ESCs towards skeletal myogenesis or neurogenesis, indicates that the effect of the mixed esters was rather specific in nature or that it did not simply enhance the overall differentiating ESC potential [71]. This view is further corroborated by a number of observations, showing that: (1) In mouse ESCs, the transcription of *GATA-4*, *Nkx-2.5*, and that of the *prodynorphin* gene itself not only was increased by HBR at the level of embryoid bodies, but they were also enhanced in puromycin-selected ESC-derived cardiomyocytes [71]; (2) the mixed ester proved effective in increasing the gene expression of the two cardiac-specific markers α-MHC and MLC-2V in puromycin selected cells [71]; (3) it enhanced the yield of MHC-positive cells together with the yield of spontaneously beating cardiomyocytes [71]; (4) HBR increased the yield of ESC-derived cardiomyocytes at a substantially higher level than the monoesters of hyaluronan with butyric (HB) or retinoic acids (HR) [71]; (5) in multi-source hMSCs, including those that had been isolated from the bone marrow, the dental pulp and term placenta, HBR lack any effect on *MyoD* gene transcription, a major conductor of skeletal myogenesis [85], and *neurogenin 1*, a neurogenic transcription factor [86]. Nevertheless, the observation that the transcriptional responses elicited by HBR in hMSCs affected genes involved in vasculogenic/angiogenic pathways [85], indicates that the action of the mixed ester besides involving the *prodynorphin* gene, encompassed a modulation of other genes playing a pivotal role in the cardiovascular system. 

The cardiogenic/vasculogenic effects induced in vitro on stem cells by HBR, could also be deployed in vivo, to afford efficient strategies of cardiac regeneration. In both small (rats) [85], and large animal models (pigs) of acute myocardial infarction [87], the transplantation of HBR-preconditioned FMhMSCs enhanced myocardial vascularization and contractility, while reducing the infarct scar size, at a significantly greater extent, as compared to the transplantation of untreated FMhMSCs [87].

Intriguingly, by the aid of magnetic resonance imaging, positron emission tomography, and immunohistochemistry, we have shown that the injection of HBR itself into infarcted rat hearts afforded a substantial cardiovascular repair, reducing the infarct size and affording a full recovery of myocardial performance, without the needs for stem cell transplantation [88]. HBR restored cardiac [18F] fluorodeoxyglucose uptake, increased capillary density, and decreased the number of apoptotic cardiomyocytes, recruiting endogenous Stro-1-positive stem cells at the infarct border zone [88]. The possibility that HBR may have acted as a synthetic intracrine in the rescue of damaged myocardium is supported by multiple layers of evidence: (1) Nuclear run-off transcription assays revealed that the transcription of *GATA-4*, *Nkx-2.5* (two cardiogenic genes) and *HGF* (a vasculogenic gene) was enhanced in nuclei that had been isolated from HBR treated hMSCs, as compared with nuclei from untreated cells [88]; (2) similarly, the transcription of *VEGF* (another vasculogenic gene) and *Pim-1* (a pro-survival gene) were increased in nuclei obtained from HBR exposed Stro-1 positive stem cells [88]; (3) nuclear run-off experiments showed that the transcription of the above-mentioned genes was not affected by direct exposure to HBR of nuclei isolated from undifferentiated ESCs or hMSCs. On the contrary, the transcription rate of the same genes was increased with superimposable time courses by nuclear exposure to butyric or retinoic acids and was additively enhanced by a mixture of the two [88,89,90]. These findings suggest that after internalization HBR may have been subjected to hydrolysis by ubiquitous intracellular esterases, unfolding an intracrine action of its own grafted moieties. In both ESCs and hMSCs, HBR differentially affected the patterning of Smad proteins, acting as major conductors in cardiogenesis, enhancing the gene and protein expression of Smad1, 3, and 4, while downregulating Smad7, with increased transcription rates of Smad4 in nuclei isolated from HBR treated cells [89]. Within this context, an intracrine pathway for HBR was supported by the observation that Smad4 binding to the *Nkx-2.5* gene resulted in being crucial for HBR mediated cardiogenesis. Chromatin immuno-precipitation experiments showed that nuclear extracts from control and HBR-treated ESCs generated an amplified product for the Smad binding element on the *Nkx-2.5* promoter, and that a significantly higher amount of amplified DNA could be generated from nuclei isolated from HBR treated ESCs, as compared with nuclei of unexposed cells [89]. Compounding the intracrine action of HBR, the injection of the mixed ester in the infarcted rat myocardium increased tissue histone H4 acetylation at the level of myocardial nuclei [88]. This result was mirrored by the observation that acetyl-H4 immunoreactivity was also enhanced at the nuclear level in rat cardiomyocytes and Stro-1 cells exposed to HBR [88] (Table 2). Thus, HBR, a molecule boosting a gene program of cardiogenic and vasculogenic commitment in vitro and in vivo, proved to be itself acting at least in part as an intracrine regulator in the same differentiating/rescuing programs.

## 4. The Use of Electromagnetic Energy to Afford Efficient Increase in Prodynorphin Gene and Dynorphin B Expression. Implications in Myocardial Cell Growth and Stem Cell Cardiogenesis

Magnetic fields (MF) have long been shown to promote behavioral changes in vivo, and modulate cell proliferation and growth factor release in vitro [91,92,93]. Since from these initial studies, the endogenous opioid peptide network emerged among the biological systems that may represent a target for MF, as revealed by the evidence that: (1) In in vivo animal models MF is able to induce analgesic effects through an opioid-mediated mechanism [94]; (2) MF can affect the spontaneous electrical activity in the brain by interfering with the action of both endogenous, or exogenously administered opioids [95]; (3) MF increase the duration of opioid-induced anesthesia in mice [96]; (4) opioid receptor subtypes were found to mediate MF-induced decrease in central cholinergic activity in the rat [97]; (5) the effect of MF on central cholinergic systems could be blocked by the opioid receptor antagonist naltrexone [98]; (6) the same antagonist was also able to antagonize the antiparkinsonian effects of picotesla range MF [99].

Nevertheless, during the period, these findings were gathered, compelling evidence that MF may affect transcriptional responses was still lacking. Based upon our observation that a dynorphinergic system was involved in cardiac contractility and cardiogenesis, and taking into account the above-reported relevance of endogenous opioids as a target biological system for MF, we decided to investigate whether this form of physical energy could be able to influence opioid peptide gene expression in adult myocardial cells. By the aid of RNAse protection analyses, we provided evidence that extremely-low frequency MF (ELF-MF) of 50 Hz, 0.8 mT^rsm^ consistently increased *prodynorphin* mRNA levels as early as after 1 h of exposure. The stimulatory effect peaked at 4 h and was still evident after 8 h of treatment [100]. Nuclear run-off transcription experiments revealed that *prodynorphin* gene transcription was increased in nuclei that had been isolated from MF exposed cardiomyocytes. Interestingly a direct exposure to ELF-MF of nuclei isolated from rat ventricular cardiac myocytes caused a transcriptional increase in the *prodynorphin* gene of similar magnitude as that observed in nuclei from exposed myocardial cells [100]. These observations proved that the MF-induced increase in *prodynorphin* gene expression was caused by a direct action on the transcriptional machinery of the nucleus, and it was not merely resulting from changes in mRNA stability or degradation. The ELF-MF action was mediated by nuclear PKC activation, but occurred independently from changes in PKC isozyme expression and enzyme translocation [100]. ELF-MF also markedly increased the intracellular levels of dynorphin B, and enhanced its secretion in the culture medium [100]. These findings for the first time demonstrated that an opioid gene was activated by cardiomyocyte exposure to electromagnetic energy and that the cell nucleus and nuclear embedded PKC were a crucial target for the ELF-MF action. We had previously shown that endorphin peptides exerted profound effects on the phosphoinositide turnover in the myocardial cells, controlling cytosolic Ca^2+^ homeostasis in both cardiac myocytes and neurons, also modulating cardiac intracellular pH and myofilament responsiveness to Ca^2+^ [41,42,43]. Whether the raise of intracellular dynorphin B elicited by ELF-MF may affect these determinants of myocardial cell function has remained so far as a suggestive hypothesis, and at the same time an intriguing challenge, owing to the complexity of the experimental design needed to address this issue. 

In the subsequent investigation, we first found that exposure of mouse ESCs to ELF-MF of 50 Hz, 0.8 mT^RSM^, triggered the expression of *GATA-4* and *Nkx-2.5*, leading to a consistent increase in the number of spontaneously beating ESC-derived cardiomyocytes, as compared with unexposed cells [101]. Such cardiogenic effect was associated with enhanced *prodynorphin* gene expression. RNase protection analyses and nuclear run-off experiments provided evidence that the ELF-MF effect was afforded by an increase of the transcription rate of the *prodynorphin* gene in isolated nuclei, raising the intracellular levels of dynorphin B [101]. Similar to spontaneous ESC cardiogenesis, the increase in *prodynorphin* gene transcription was also associated with an increase in dynorphin B secretion in the culture medium [101]. These findings indicate that ESC exposure to electromagnetic energy was able to activate a complex circuitry linking *prodynorphin* gene transcription and cardiogenesis through the intracrine/autocrine signaling of a bioactive product of the gene itself.

It may be worthy to note that with the investigated type of ELF-MF we only obtained a cardiogenic commitment from the exposed ESCs, without significant differentiating outcomes towards non-myocardial lineages [101]; thus, ruling out the possibility that the observed cardiogenic action may simply be part of a broader, generalized transcriptional response, or it may be resulting from non-specific changes in protein degradation.

More recently, we used a different exposure technology to convey MF to both mouse ESCs and hMSCs, based upon cell stimulation with a radiofrequency of 2.4 GHz emitted by a radioelectric asymmetric conveyer (REAC) [102]. The REAC technology has the innovative feature of generating an extremely weak microwave emission, being effective at emitter power levels as low as approximately 2 mW. The REAC action is independent of the radiofrequency emission used. We used only the frequencies of 2.4 and 5.8 GHz, the most widespread and authorized worldwide (i.e., Wi-Fi). The particular physic connection between the apparatus and the cell culture or patient’s tissues, rather than emitted radiofrequency, represents the hallmark of the REAC technology. Such a connection is afforded via the asymmetric conveyer probe [102,103]. This probe is, therefore, a part of an innovative method and device enabling the emitted frequency to interact without depth limits with cultured cells in vitro or with the body tissues in vivo. Within the biological target, this interaction elicits radiofrequency-induced microcurrents, varying on the bases to the molecular features of targeted tissues or cells. Merging of such radiofrequency-induced microcurrents ensues in a resulting microcurrent, generated by the target itself. This resulting microcurrent, concentrated by the asymmetric conveyer probe, appears to be responsible for the observed biological responses and therapeutic effects [103,104]. With the REAC technology, we succeeded in optimizing pluripotency and multipotency in mouse ESCs and hMSCs isolated from the adipose tissue (AD-hMSCs), respectively, modulating the transcription of stemness genes and increasing the yield of cells differentiating into cardiac, neural, skeletal and myogenic lineages [102,105]. These findings indicate that changing the MF frequency and the modality of MF interaction with the target cells would result in a broader spectrum of transcriptional responses and in a multilineage commitment of the exposed stem cells. While the activation of neurogenesis and skeletal myogenesis involved the overexpression of *neurogenin-1* and *MyoD*, respectively, the enhancement of GATA-4 and Nkx-2.5 elicited by REAC during the cardiogenic commitment of both ESCs and adult stem cells was still associated with an increase in *prodynorphin* gene transcription, as it was observed following ESC exposure to ELF-MF [102,105]. 

These observations indicate that, while the transcriptional responses primed by REAC were placed within a wider context of cell signaling networks, the cardiogenic counterpart of the REAC action retained similar features as those that were uniquely elicited by ELF-MF (which did not affect ESC commitment outside of a cardiogenic pathway) [101]. Moreover, the results yielded following stem cell exposure to the REAC technology, further confirmed the cardiogenic role of the *prodynorphin* gene, and the feasibility of using a physical stimulation to activate an endorphinergic system and its related signaling, even within a frame of enhanced pluri-/multi-potency expression. This view was further inferred from intriguing results showing that the exposure to REAC-conveyed radioelectric fields afforded a remarkable commitment toward cardiac, neuronal, and skeletal muscle lineages even in human skin fibroblasts [106]. The REAC action was associated with a biphasic effect on a number of stemness-related genes, leading to an early transcriptional increase of *Oct4*, *Sox2*, *c-Myc*, *Nanog*, and *Klf4* within 6–20 h, followed by downregulation at later times [106]. This observation indicates that the REAC action bypassed a persistent reprogramming toward an induced pluripotent stem cell-like state, avoiding the extremely low differentiation efficiency and the risk of tumorigenic drift associated with cell reprogramming with viral vector-mediated approaches. The increase in *MyoD* and *neurogenin-1* transcription observed during the skeletal myogenesis and neurogenesis elicited by REAC led to the expression of the corresponding tissue-restricted marker proteins, MyoD and β3-tubulin, as assessed by confocal microscopy analysis [106]. The transformation of human skin fibroblasts into cardiac-like cells expressing α-MHC and SA, was again mediated by the transcriptional activation of a cardiogenic program, encompassing a consistent increase in the transcription of the *prodynorphin* gene, together with the overexpression of other remarkable cardiogenic transcripts, including *Mef2c*, *Tbx5*, *GATA-4*, and *Nkx-2.5* [106]. These findings also showed for the first time the chance of using physical energy to frame the activation of a conductor of cardiogenesis, like the *prodynorphin* gene, within the context of a pluripotentiality expression in human adult somatic cells (Table 3). Compounding the spectrum of the biological actions of the REAC technology, we found that it was able to counteract and even reverse stem cell aging through the rescue of both telomerase-dependent and -independent pathways [107,108,109]. These effects involved a substantial contribution from intracrine hyaluronan-related mechanisms, since the chemical abrogation of endogenous type-2 hyaluronan synthase remarkably counteracted the antiaging effect of REAC [110]. Whether the intracellular hyaluronan signaling may also encompass the deployment of intracrine endorphinergic paths remains to be established, and it is the subject for our ongoing investigation.

## 5. Conclusions and Future Perspective

Collectively, the findings reported here show the participation of dynorphin-mediated intracrine positive feedback loops in the differentiation of stem cells along a cardiac lineage and further suggest that dynorphin signaling interacts with targeted transcription factors, possibly through the upregulation of other intracrine loops. For example, the recruitment of *Nkx-2.5* and *GATA-4* raises the possibility that they too could behave in an intracrine fashion. Feed-forward mechanisms are executed through the ability of dynorphin B of acting on its coupled receptor and signaling in isolated nuclei to stimulate the transcription of its own coding gene, as well as the transcription of homeodomain and zinc-finger transcription factors. Analysis of these circuitries suggests the establishment of a positive feedback loop, intervening in the timely amplification and synchronization of multiple intracrinergic systems, since transcription factors, including zinc-fingers and the homeodomains, have been found to be exchanged among cells through the exosomal route and act intracellularly after internalization, fulfilling the pattern requirement for being considered as intracrines. The intracrine involvement in stem cell commitment prompts the use of intracrine peptides for reprogramming (stem) cells to a pluripotency state, thereby obviating the needs for viral transfection and possible secondary neoplastic transformation. Indeed, it is becoming clear that the therapeutic effects elicited by stem cells are multifaceted, and, in large part, they result from actions other than their proliferation and direct differentiation into cells composing the adult tissues. Among these other actions of tissue stem cells is included the elaboration of a variety of cytokines and growth factors. The possibility of intracrine patterning played by these trophic mediators in modulating the fate of stem cells and their rescuing potential appears to be worthy of exploration. We are currently becoming aware of the fact that exposure to physical energies, like MF of different frequencies and modalities of conveying, and possibly other physical stimuli, including mechanical vibration and light, can be exploited to variably modulate the transcription of genes essential in crucial fate decisions of stem cells. This approach may offer the chance of controlling intracrine pathways in a precise way without the use of viral vector-mediated tools or cumbersome chemistry, not only in vitro, but even in vivo. In fact, based upon the diffusive features of these physical energies, we are envisioning the perspective of targeting human stem cells in place, where they already are, resident in all the tissues of the human body, affording the modulation of multifaceted pathways, as those controlled by intracrine molecules, without the need of stem cell or tissue transplantation, boosting our inherent self-healing ability.

## Figures and Tables

**Table 1:** Effects of different agents on cardiac function and commitment.

Stimulus	Species	Cell Type	Biological Effects	Ref.
KCl^1^ 60 mM (for 4 or 24 h of treatment)	Rat	Ventricular Cardiomyocytes	Increased expression of the *prodynorphin* gene Increase of dynorphin B At the intracellular level and in the culture medium	[52]
PMA^2^ 100 nM (from 1 to 24 h of treatment)	Rat	Cardiomyocytes and nuclei isolated from cardiomyocytes	Activation of the nuclear protein kinases C-δ and -ε Subsequent increased expression of the *prodynorphin* gene Increase of dynorphin B At the intracellular level and in the culture medium	[53]
DMSO^3^ 1% (for 2 or 4 days of treatment)	Mouse	P19 ESCs^4^	Increased expression of the *prodynorphin* gene Following an increase of *GATA-4* and *Nkx-2.5* expression Increase of dynorphin B At the intracellular level and in the culture medium Counteracted by opioid receptor antagonism or targeted inhibition of *prodynorphin* gene expression	[55]

^1^ Potassium chloride; ^2^ Phorbol 12-myristate 13-acetate; ^3^ dimethyl sulfoxide; ^4^ embryonic stem cells.

**Table 2:** Hyaluronan mixed esters of butyric and retinoic acids (HBR) studies for cardiac commitment and repair.

Stimulus	Species	Cell Type	Biological Effects	Ref.
HBR^1^ 0.75 mg/mL for five days of treatment	Mouse	ESCs	Overexpression of the *prodynorphin* gene Increase in *GATA-4* and *Nkx-2.5* transcription Increase of dynorphin B at the intracellular level and in the culture medium Increase of the number of spontaneously beating myocardial cells Expression of α-MHC and MLC-2V in spontaneously beating cardiomyocytes Absence of effect on skeletal myogenic or neurogenic markers	[71]
HBR 1.5 mg/mL for seven days of treatment (fourteen days for analyses of cardiac markers)	Human	MSCs^2^ isolated from different sources, including the bone marrow, dental pulp, and FM^6^	Improvement of *VEGF*^3^, *KDR*^4^, and *HGF*^5^ gene expression Increase of VEGF and HGF secretion Increase in *GATA-4* and *Nkx-2.5* transcription Expression of cardiac markers Absence of effect on skeletal myogenic or neurogenic markers	[85]
Transplantation of HBR-preconditioned FMhMSCs (1.5 mg/mL for 14 days) in animals affected by acute myocardial infarction	Rat and Pig	Cardiomyocytes	Enhancement of myocardial vascularization and contractility Reduction of the infarct scar size	[85,87]
HBR injection (0.2 mg/100 g of rat weight) in heart affected by acute myocardial infarction	Rat	Cardiomyocytes	Presence of cardiovascular repair, reducing the infarct size and affording a full recovery of myocardial performance Increase of capillary density, and decrease of the number of apoptotic cardiomyocytes Increase of histone H4 acetylation at the level of myocardial nuclei	[88]
HBR 2 mg/mL (from 1 to 10 days of treatment)	Human	MSCs	Enhancement of Smad1, 3, and 4 gene and protein expression Downregulation of Smad7 Increase of transcription rates of Smad4 in nuclei isolated from HBR treated cells correlated to *Nkx-2.5* expression	[89,90]

^1^ Hyaluronan mixed esters of butyric and retinoic acids; ^2^ mesenchymal stem cells; ^3^ vascular endothelial growth factor; ^4^ kinase insert domain receptor; ^5^ hepatocyte growth factor; ^6^ fetal membranes of term placenta.

**Table 3:** Magnetic fields (MF) studies and cardiac commitment.

Stimulus	Species	Cell Type	Biological Effects	Ref.
ELF-MF^1^ of 50 Hz, 0.8 mT^RSM^ (for 4 h of treatment)	rat	Nuclei isolated from ventricular cardiac myocytes or intact cells	Increase in *prodynorphin* gene transcription Increase in intracellular levels of dynorphin B Enhancement in dynorphin B secretion in the culture medium	[100]
ELF-MF of 50 Hz, 0.8 mT^RSM^(for three and ten days of treatment)	mouse	ESCs	Induction of *GATA-4* and *Nkx-2.5* gene expression Increase in the number of spontaneously beating ESC-derived cardiomyocytes Increase in the transcription rate of the *prodynorphin* gene Increase in intracellular levels of dynorphin B Enhancement in dynorphin B secretion in the culture medium	[101]
Radiofrequency of 2.4 GHz emitted by REAC^2^ (for treatment details see Ref.)	mouse and human	ESCs and AD-MSCs	Modulation in the transcription of stemness genes Increase in the yield of cells differentiating into cardiac, neural, skeletal and myogenic lineages Association of cardiac differentiation with *GATA-4*, *Nkx-2.*5 and *prodynorphin* gene transcription	[102,103,104,105]
Radiofrequency of 2.4 GHz emitted by REAC (from 2 to 72 h of treatment and 72 h of treatment + four or seven days of recovery)	human	Dermal skin fibroblasts	Early transcriptional increase of *Oct4*, *Sox2*, *c-Myc*, *Nanog*, and *Klf4*, stemness genes followed by their downregulation Increase in the yield of cells differentiating into cardiac, neural and myogenic lineages Association of cardiac differentiation with: Expression of *Mef2c*, *Tbx5*, *GATA-*4 and *Nkx-2.5* genes; expression of MHC and SA proteins; increase in the expression of *prodynorphin* gene	[106]

^1^ extremely-low frequency magnetic fields; ^2^ radioelectric asymmetric conveyer.

## Abbreviations

ACE	Angiotensin Converting Enzyme
AD	Adipose tissue
CPCs	Cardiac progenitor cells
DMSO	Dimethyl sulfoxide
ELF-MF	Extremely-low frequency magnetic fields
ESCs	Embryonic stem cells
FGF1	Fibroblast growth factor 1
FM	Fetal membranes
HBR	Hyaluronan mixed esters of butyric and retinoic acids
HEL	Human erythroleukemia cells
HGF	Hepatocyte growth factor
hMSCs	Human mesenchymal stem cells
HSCs	Hematopoietic stem cells
Ins(1,3,4,5)P4	Inositol 1,3,4,5-tetrakisphosphate
Ins(1,4,5)P3	Inositol 1,4,5-trisphosphate
KCl	Potassium chloride
KDR	Kinase insert domain receptor
MF	Magnetic fields
MHC	Myosin heavy chain
MKs	megakaryocytes
MLC	Myosin light chain
PKC	Protein kinase C
PMA	Phorbol 12-myristate 13-acetate
REAC	Radioelectric asymmetric conveyer
SA	Sarcomeric actinin
VEGF	Vascular endothelial growth factor
